# Supplementary material for: Osseosurface electronics—thin, wireless, battery-free and multimodal musculoskeletal biointerfaces
Source: Nat Commun. 2021 Nov 18;12:6707. doi: 10.1038/s41467-021-27003-2 (PMC8602388; doi:10.1038/s41467-021-27003-2)
Supplement: Supplementary file 1 — Supplementary information. [file 41467_2021_27003_MOESM1_ESM.docx]

**Supplementary Information**

**Osseosurface electronics – Thin, wireless, battery-free and multimodal musculoskeletal biointerfaces**

Le Cai^1+^, Alex Burton^1+^, David A. Gonzales^2^, Kevin Albert Kasper^1^, Amirhossein Azami^1^, Roberto Peralta^3^, Megan Johnson^1^, Jakob A. Bakall Loewgren^1^, Efren Barron Villalobos^2^, Ethan C. Ross^1^, John A. Szivek^1,2^, David S. Margolis^1,2^*, Philipp Gutruf^1,4^*

^1^ Department of Biomedical Engineering, University of Arizona, Tucson, AZ 85721

^2^ Department of Orthopaedic Surgery and Arizona Arthritis Center, University of Arizona, AZ 85721

^3^ Department of Aerospace and Mechanical Engineering, University of Arizona, Tucson, AZ 85721

^4^ Departments of Electrical and Computer Engineering, BIO5 Institute, Neuroscience GIDP, University of Arizona, 85721.

^+^These authors contributed equally

* These authors jointly supervised this work

Correspondence should be addressed to:

David S. Margolis (dsm@arizona.edu)

Philipp Gutruf (pgutruf@email.arizona.edu)


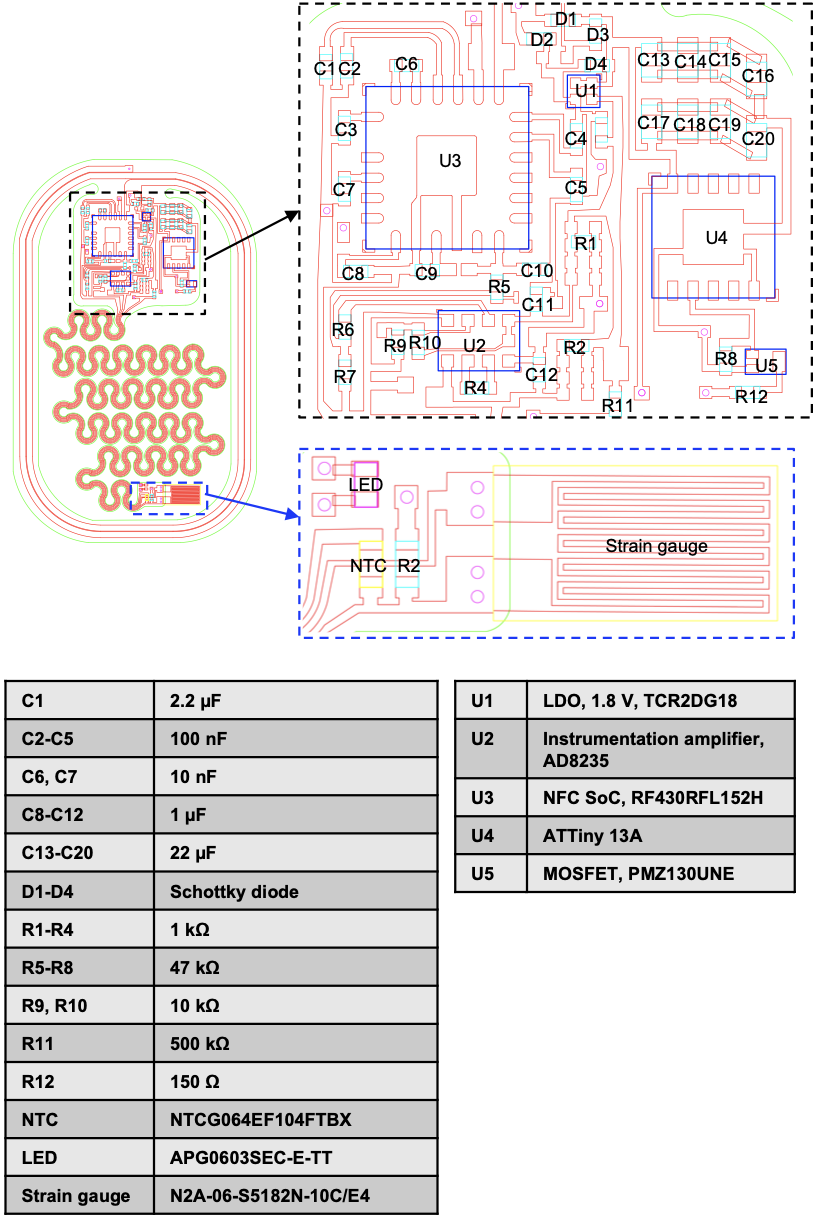


**Supplementary Figure 1.** Circuit layout including the main electronics and the biointerface, and list of components used.


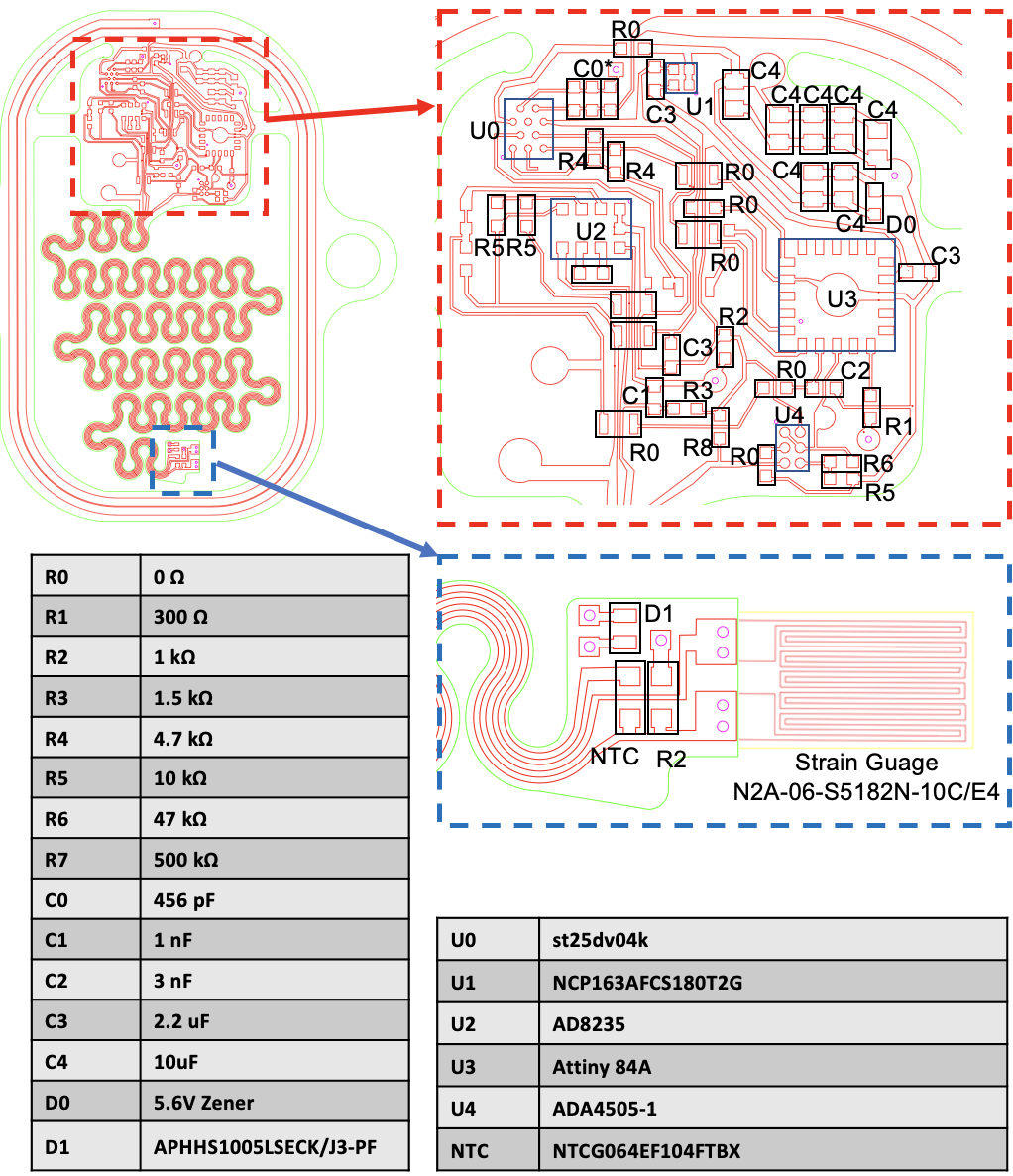


**Supplementary Figure 2.** Circuit layout including the main electronics and the bio-interface, and a list of components for the ST25DV04k NFC chip with dedicated power harvesting.


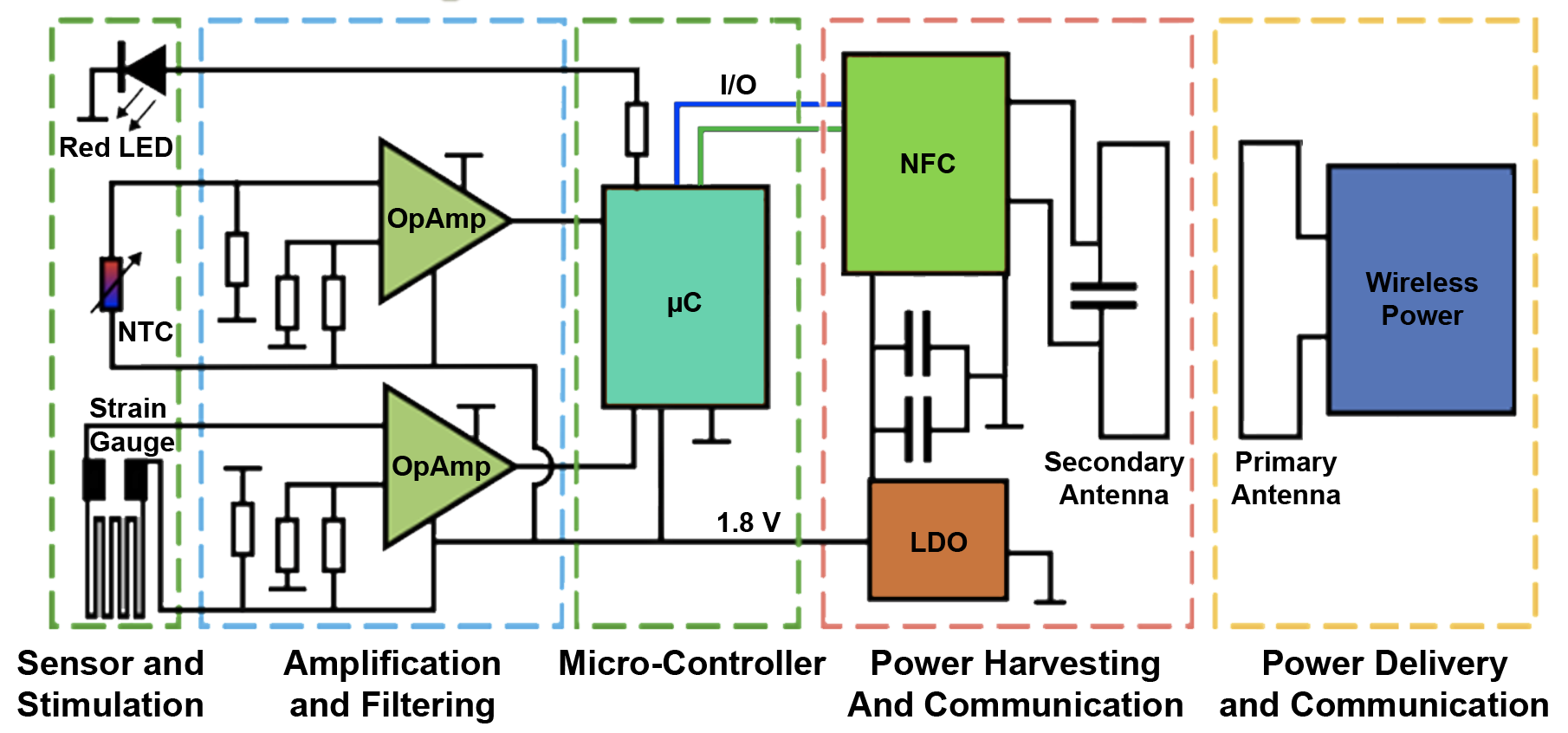


**Supplementary Figure 3.** Block diagram for devices using the ST25DV04K NFC chip.

**
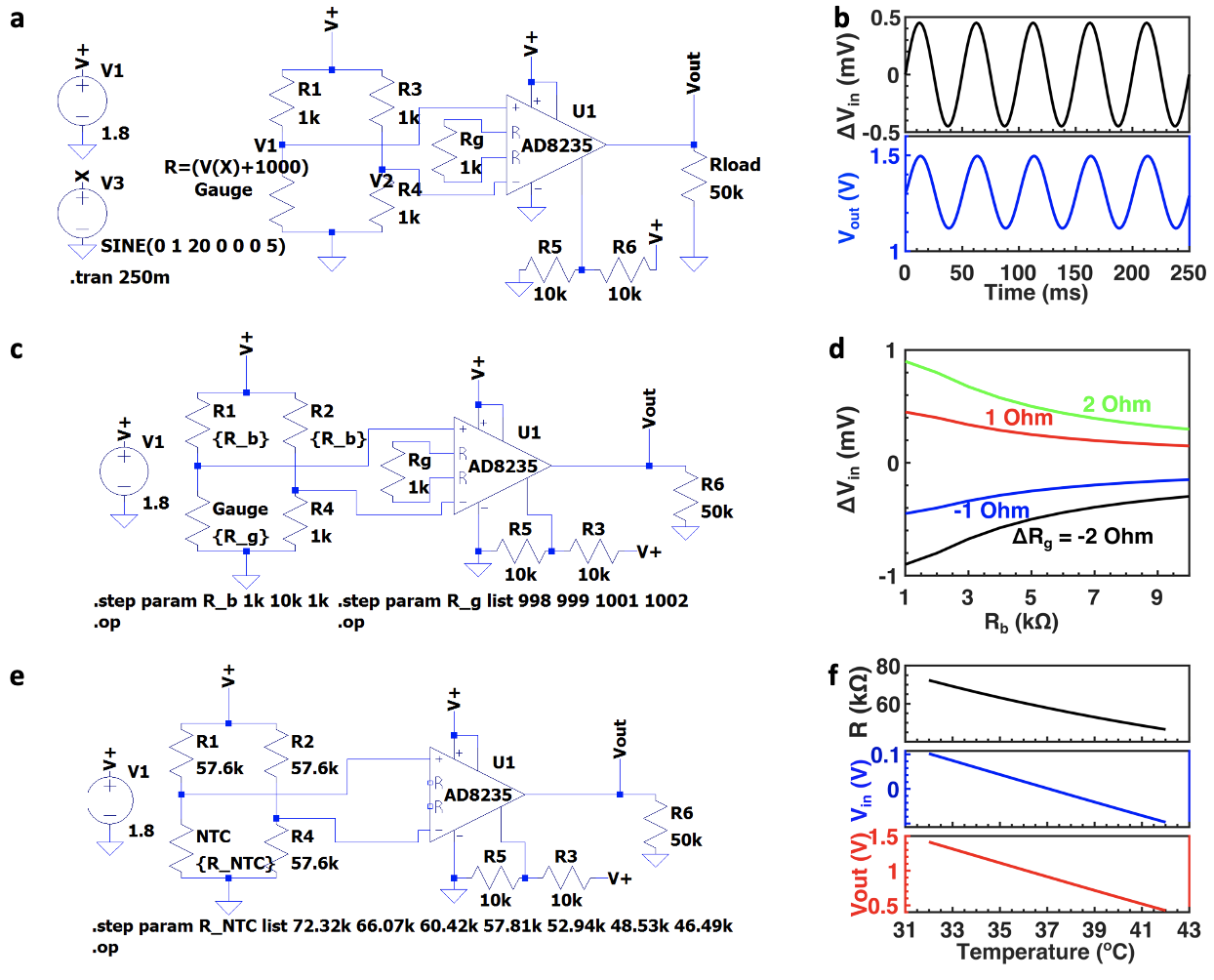
 Supplementary Figure 4. Circuit simulation of the analog front end of strain sensor (a-d) and thermography (e-f). a-b** Output voltages from the Wheatstone bridge and the instrumentation amplifier as the resistance of the strain gauge varies from 999 Ω to 1001 Ω (corresponding to strain from -500 με to 500 με) following a sinusoidal waveform (20 Hz): circuit diagram with simulation conditions (**a**) and simulation results (**b**). **c-d** Output voltage from the Wheatstone bridge as the bridge resistors, R_1_ and R_2_, vary from 1 kΩ to 10 kΩ for strain of 500 με and 1000 με: circuit diagram with simulation conditions (**c**) and simulation results (**d**). **e-f** Output voltages from the Wheatstone bridge and the instrumentation amplifier as the temperature varies from 32 °C to 42 °C: circuit diagram with simulation conditions (**e**) and simulation results (**f**).

**
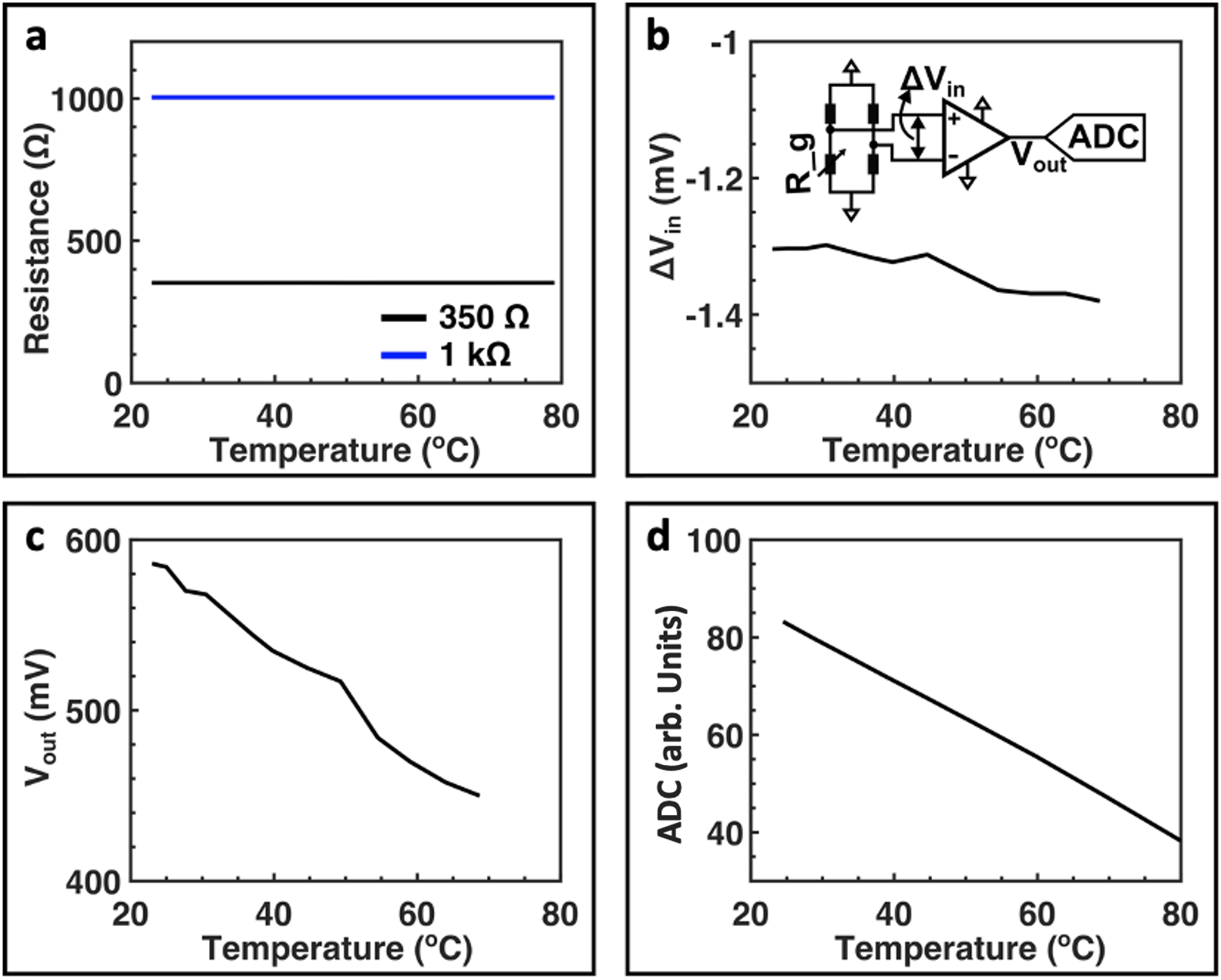
**

**Supplementary Figure 5. Thermal stability of the strain sensor analog front–end. a** Resistance of the 350 Ω gauge and 1000 Ω gauge as functions of temperature. **b** Output voltage from the Wheatstone bridge as a function of temperature. Inset, circuit diagram. **c** Output voltage from the instrumentation amplifier as a function of temperature. **d** Wirelessly recorded ADC reading as a function of temperature.

**
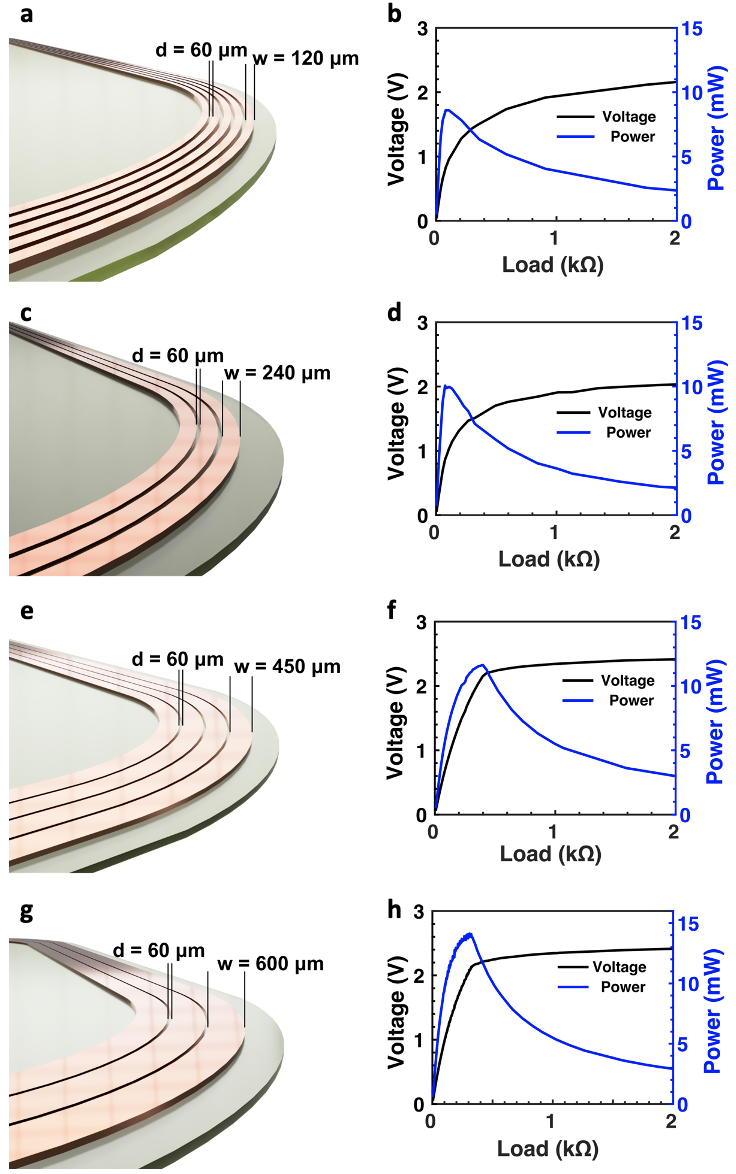
**

**Supplementary Figure 6. Optimization of the secondary antenna.** 3D rendering of secondary antennas with various geometries **(a, c, e, g)** and the corresponding power harvesting capabilities with NFC SoC **(b, d, f, h).**

**
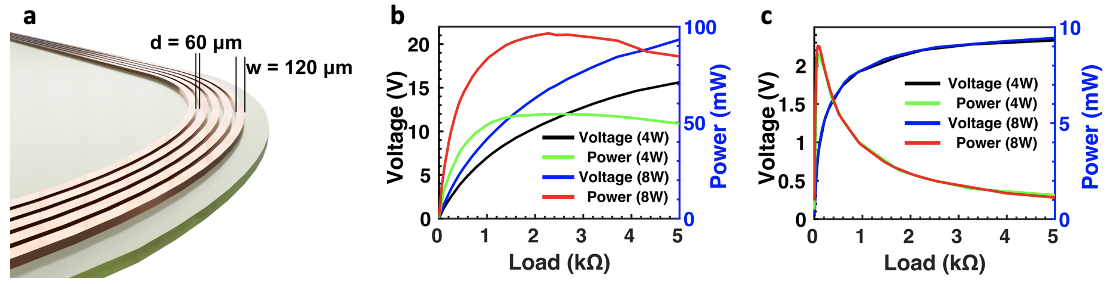
Supplementary Figure 7. Comparison of the power harvesting capability of devices with and without NFC SoC. a** 3D rendering of the secondary antenna with a copper trace width of 120 μm and inter-trace distance of 60 μm. **b** Power harvesting capability, i.e. rectified voltage and harvested power as functions of electrical load, of the device without NFC SoC. **c** Power harvesting capability of the device with NFC SoC, showing the voltage clamping by the NFC SoC.

**
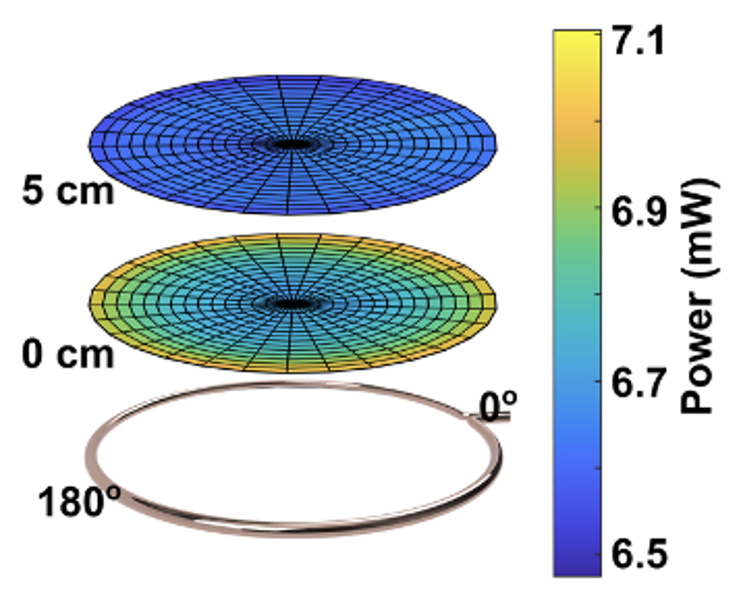
**

**Supplementary Figure 8.** Spatial distribution the harvested power with the large animal device at a load of 900 Ω from a handheld primary antenna (20 cm).


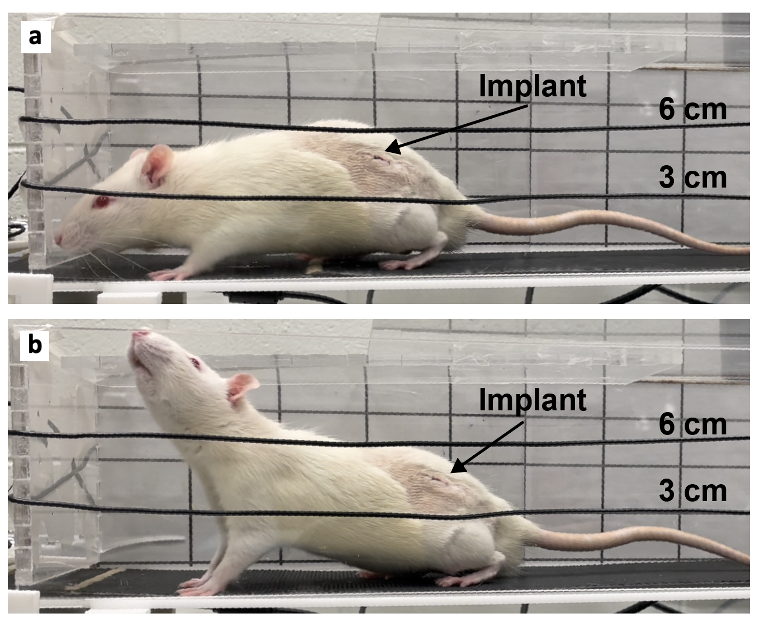


**Supplementary Figure 9. Photograph of animal postures and height of device body relative to antenna position. a** Photograph of animal walking on treadmill. **b** Photograph of animal during head raising.

**
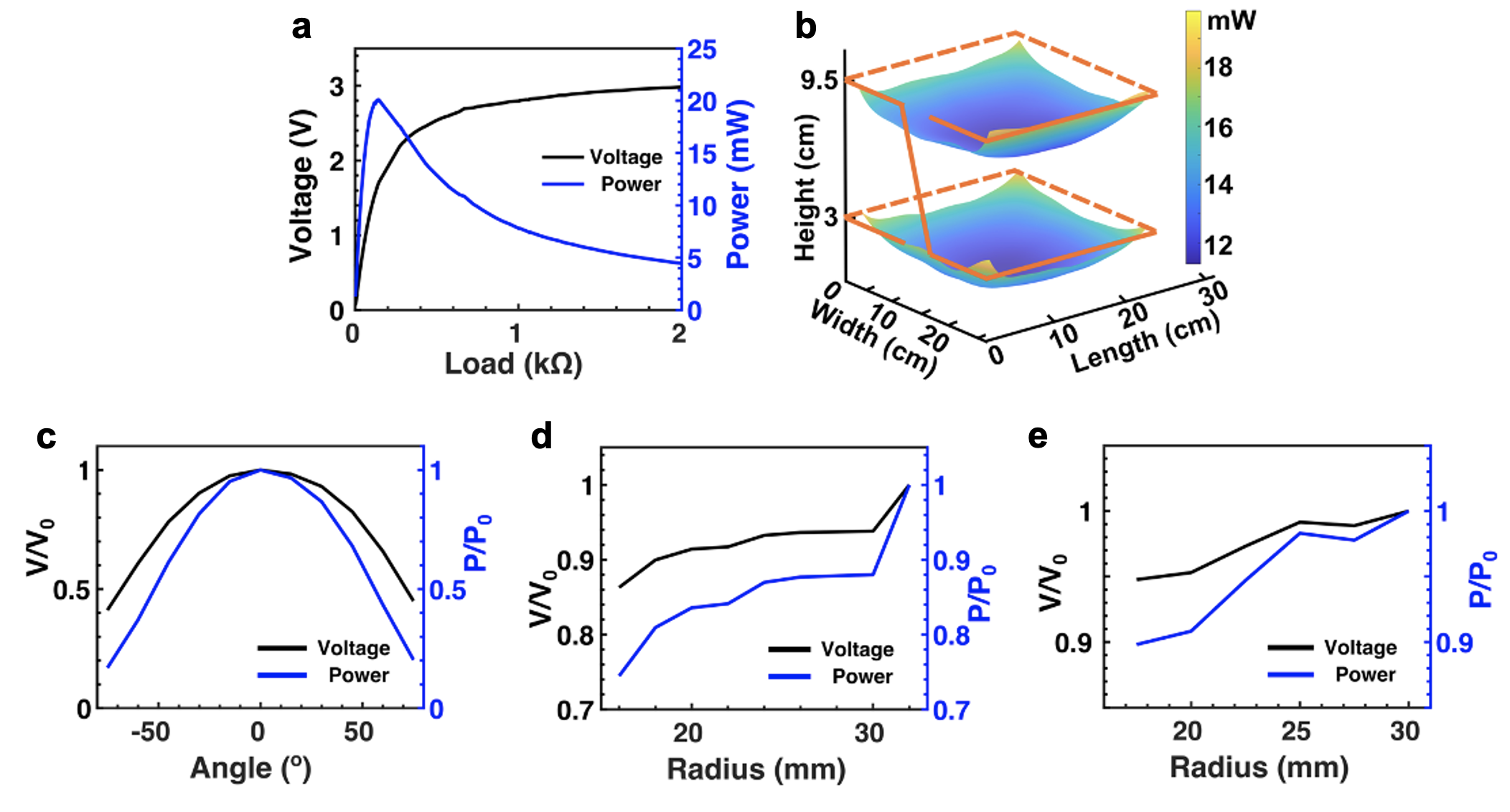
Supplementary Figure 10. Power harvesting performance of the secondary antenna in various test arenas. a-b** Power harvesting capability **(a)** and spatial distribution of harvested power **(b)** of the small animal device with a 2-turn 33 cm x 26 cm primary antenna that encloses the rat home cage. **c-e** Normalized power harvesting capability as functions of rotation angle (**c**), bending radius (**d**) and 3D bending radius (**e**) of the device.

**
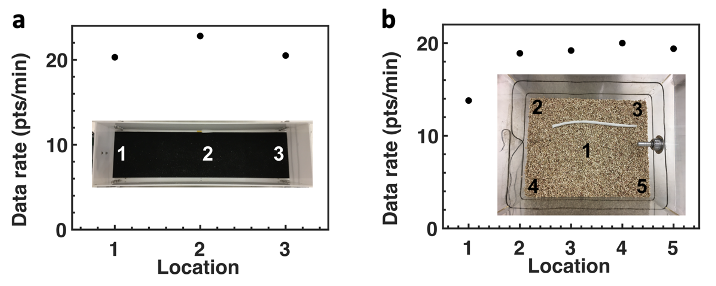
**

**Supplementary Figure 11. Wireless communication performance. a** Data rate of the rat device in various locations in the treadmill cage (2-turn, 45 cm x 12 cm). **b** Data rate of the rat device in various locations in the home cage (2-turn, 33 cm x 26 cm).

**
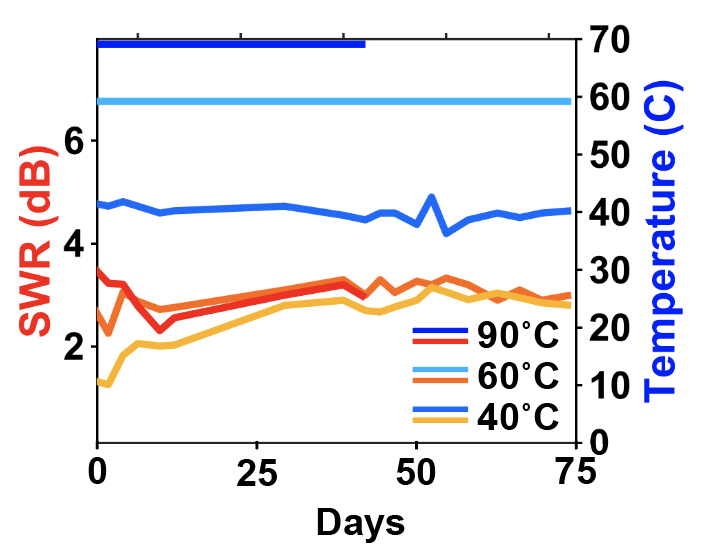
**

**Supplementary Figure 12**. Accelerated rate testing at 90˚C, 60˚C, and 40˚C in PBS, testing for wireless communication function, optical stimulation function, standing wave ratio of antenna (Red, Orange, Yellow), and wireless recording of temperature (Navy, Light blue, Blue).


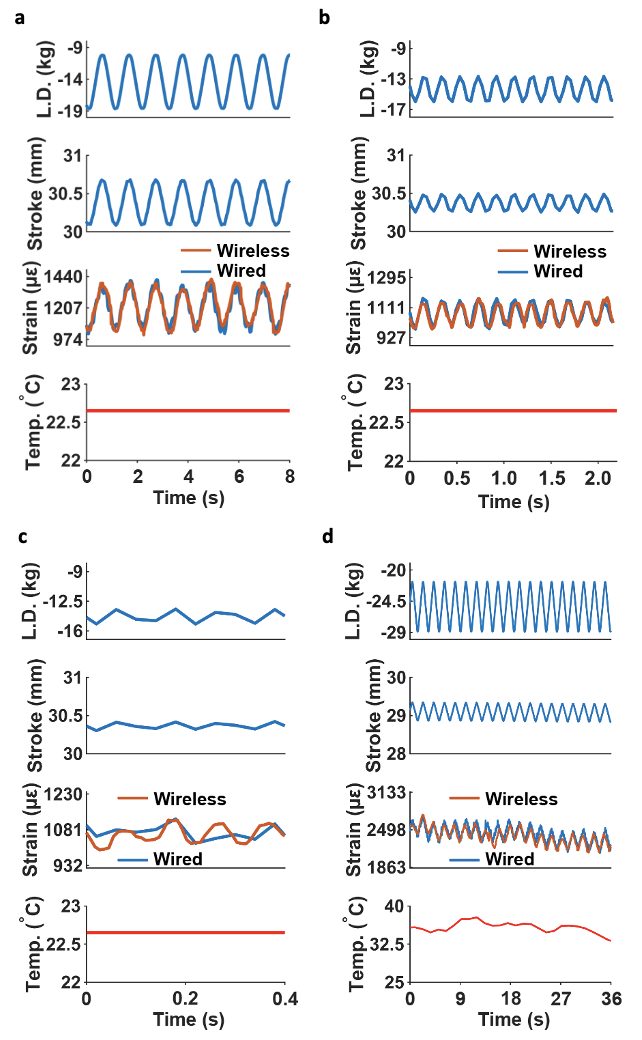


**Supplementary Figure 13. Electromechanical benchtop testing of wireless device on sheep femur. a** 1 Hz sine load cycle. **b** 5 Hz sine load cycle. **c** 10 Hz sine load cycle. **d** 1 Hz triangle load cycle with active convective heating is heat gun of the bone and device to match average body temperatures in a rat.

**
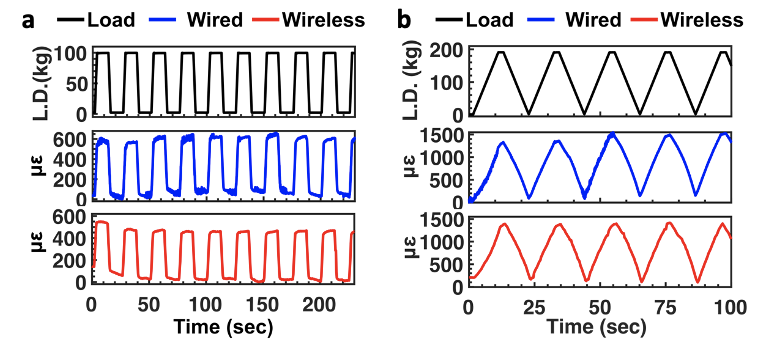
**

**Supplementary Figure 14. Bench top tests of the wireless strain sensor attached on a sheep femur specimen. a-b** Load, strain recorded by the wired sensor and wireless device when the femur is subjected to cyclic loads with square (**a**) and triangle (**b**) waveforms.

**
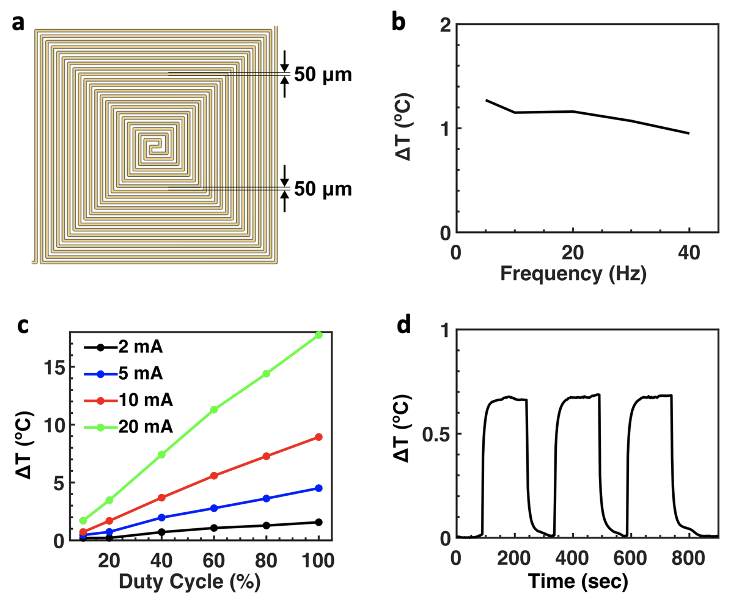
**

**Supplementary Figure 15. Bench top characterization of the wireless thermography. a** Design layout of the micro-heater. **b** Thermal impact of the μ-ILED in PBS as a function of operation frequency (duty cycle ~50%, optical power ~100 mW mm^-2^) recorded wirelessly by the co-located NTC thermistor. **c** Thermal impact of an LED as functions of driving current and duty cycle (frequency ~20 Hz) in air measured wirelessly by the co-located NTC thermistor. **d** Temporal profile of the thermal impact of an LED powered by the rectified voltage and controlled by a microcontroller with a duty cycle of 75% in PBS.

**
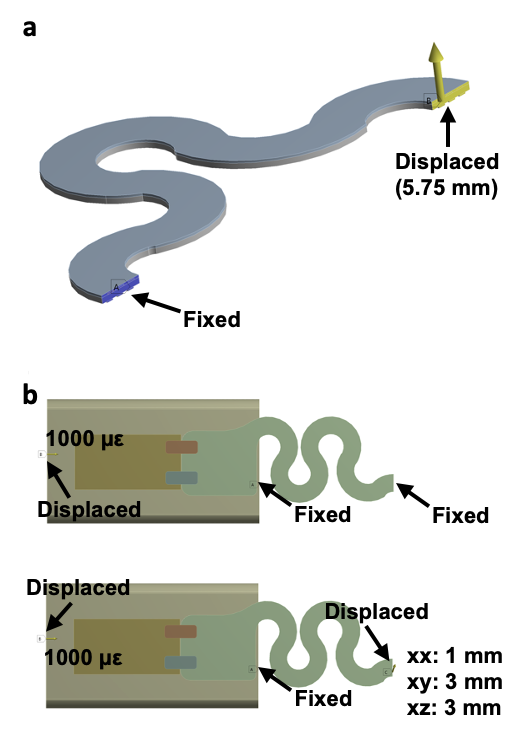
**

**Supplementary Figure 16. FEA models and simulation conditions for the mechanical simulation. a** Simulation of the strain in the copper traces while the serpentine interconnects are stretched. **b** Simulation of the mechanical isolation of the strain gauge provided by the serpentine interconnects.

**
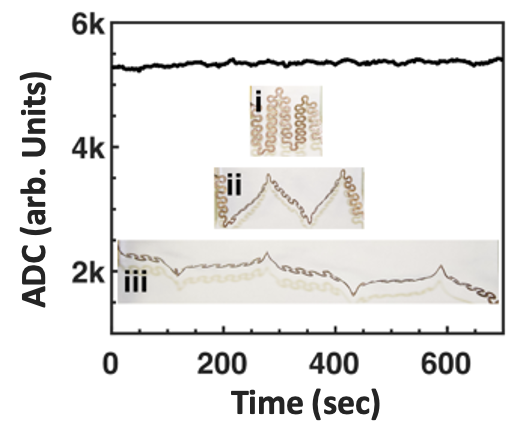
**

**Supplementary Figure 17. Stability of serpentine interconnects.** Wirelessly recorded ADC reading from the strain sensor (gain ~ 215, strain gauge unloaded) while the serpentine interconnects are cyclically stretched from 2 cm to 7 cm. Insets, photographs of the serpentine interconnects being stretched to different length: i. relaxed (2 cm); ii. 4 cm; iii. 7 cm**.**


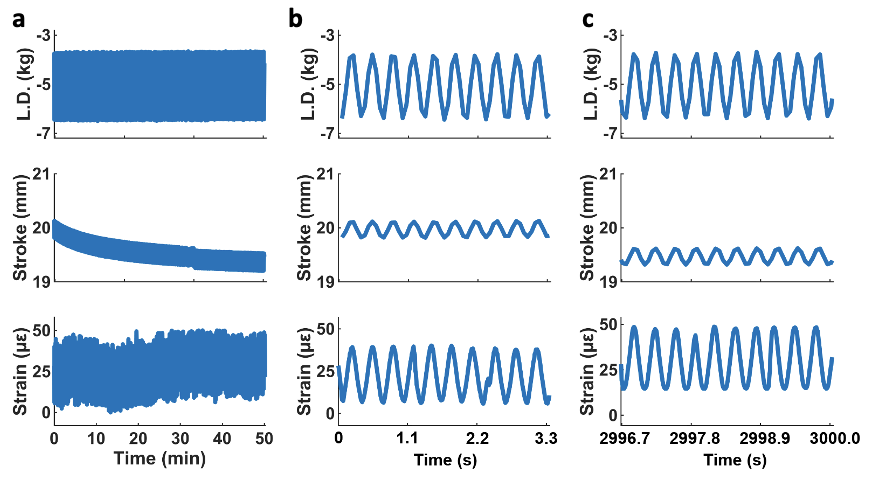


**Supplementary Figure 18. Extended servo-hydraulic materials testing. a** 10,000 cycle testing on an MTS (Series 810, MTS Systems Corporation). **b** First 10 cycles of testing procedure. **c** Last 10 cycles of testing.

**
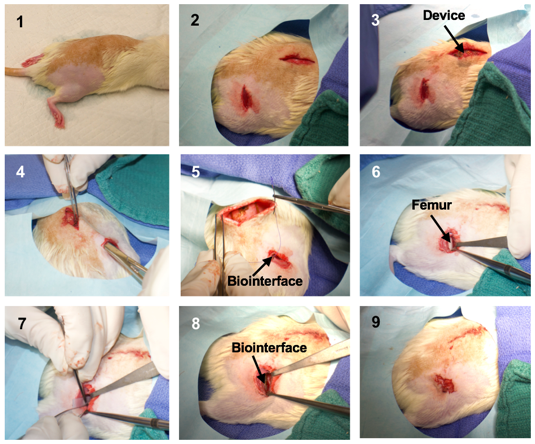
Supplementary Figure 19. Step-by-step images of the procedures to implant the osseosurface device in rats.** 1. Rat under anesthesia with fur shaved on the back and hind limb; 2. Incision made into the skin on the back and the limb; 3. Device inserted subcutaneously on the back; 4. Bio-interface being tunneled subcutaneously to the hind limb; 5. Closing the skin on the back by suturing; 6. Femur exposed; 7. Attaching the biointerface on the femur; 8. Biointerface attached; 9. Incision on the limb closed by suturing.


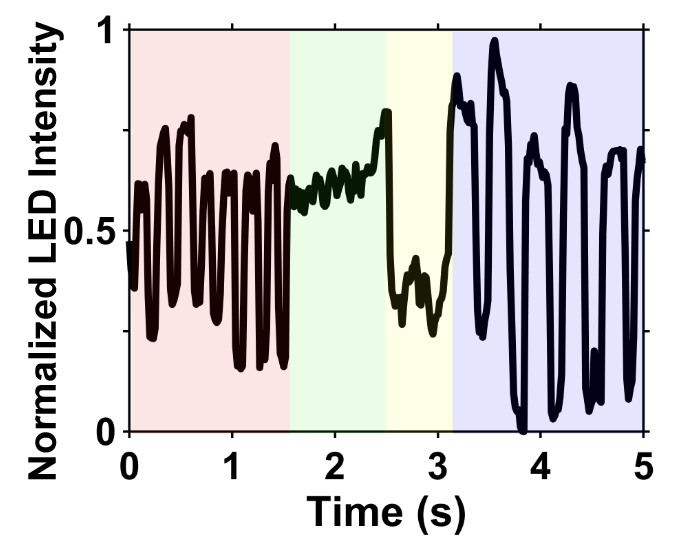


**Supplementary Figure 20.** Tracked red light intensity of optogenetic stimulation while implanted with control of frequency and duty cycle: 9 Hz, 50% (red); 9 Hz, 100% (green); Off (yellow); 5 Hz, 75% (blue).


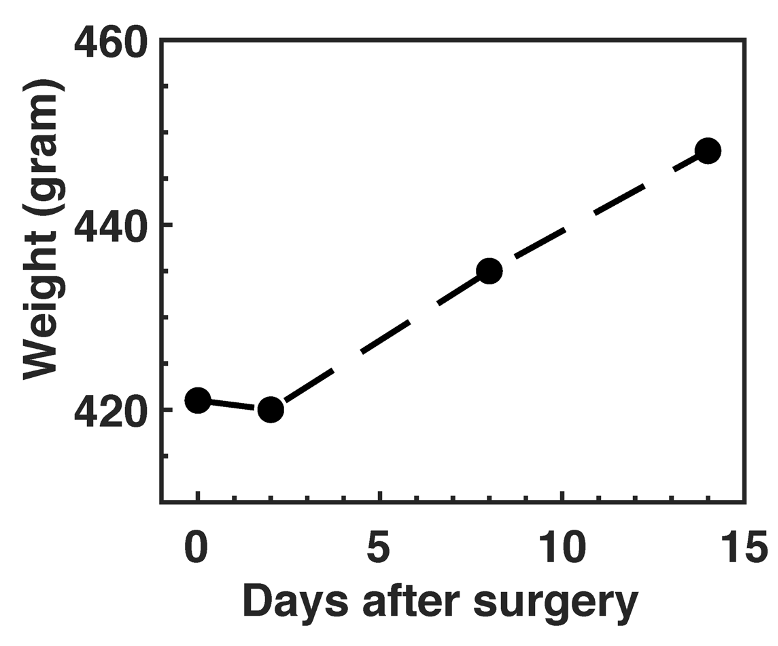


**Supplementary Figure 21.** Weight of the subject as a function of time post-surgery.

**
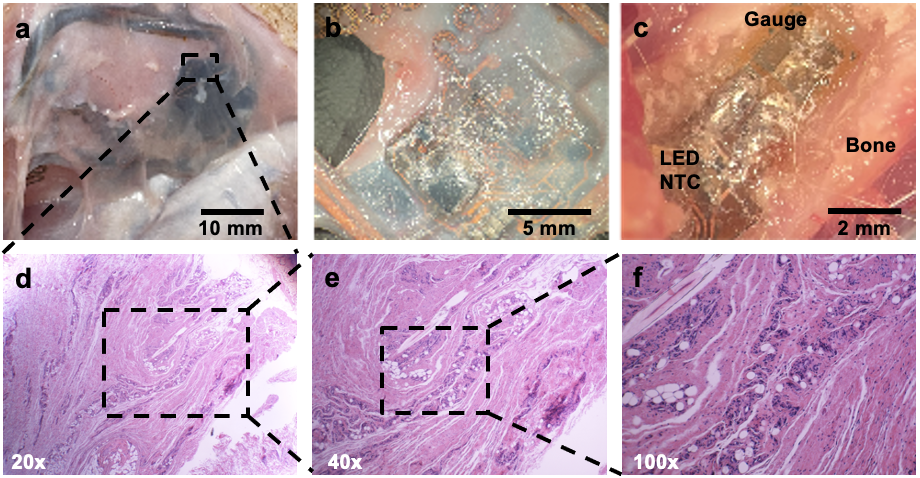
**

**Supplementary Figure 22. Tissue analysis surrounding the device after explantation. a** Photograph of tissue growth around device. **b** Photograph of tissue under microscope. **c** Photograph of strain gauge glued to the bone. **d** Histology of tissue sample surrounding device with 20x magnification. **e** Histology of tissue sample surrounding device with 40x magnification. **f** Histology of tissue sample surrounding device with 100x magnification.

**
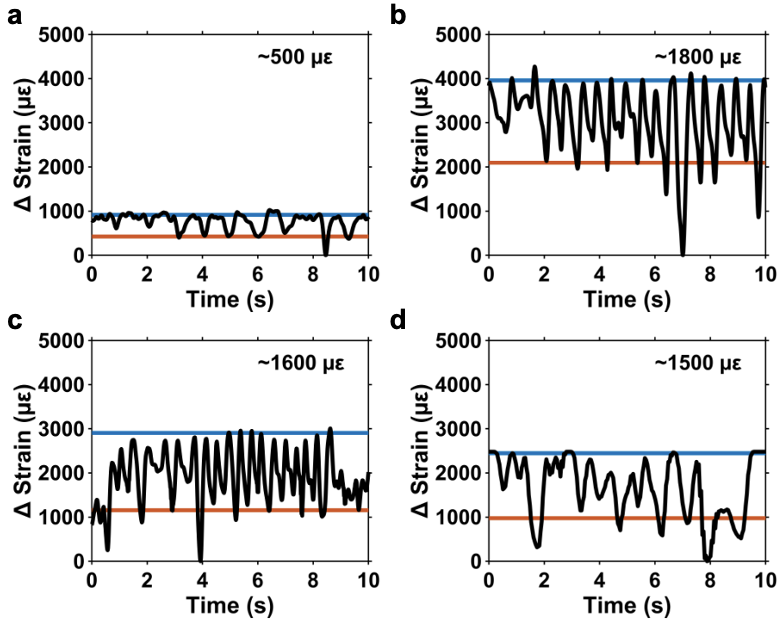
**

**Supplementary Figure 23. Strain gauge data collected over 2 weeks post implantation** **with the average high (blue) and low (red) peaks in each recording session. a** Stain recordings 0 days post-surgery. **b** 1 day post-surgery. **c** 7 days post-surgery. **d** 17 days post-surgery.

**
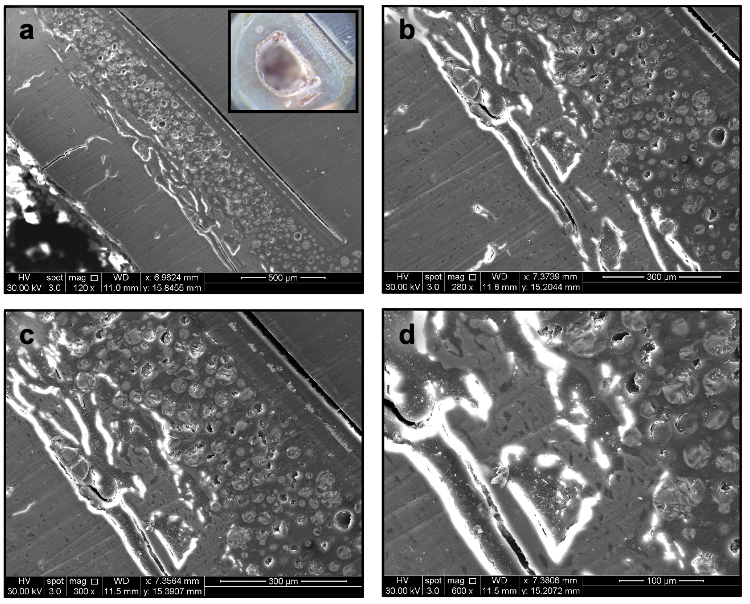
**

**Supplementary Figure 24. Additional SEM cross section images of CPC bone bonding. a** SEM cross section of strain gauge bonded to the bone using CPC particles with a magnification of 120x and a photograph image inset. **b** SEM cross section with a magnification of 280x, **c** 300x, and **d** 600x.

**
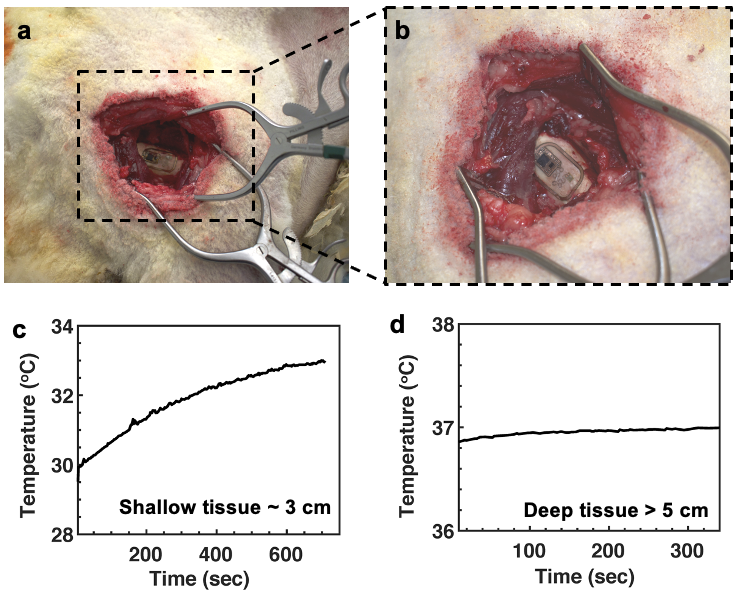
**

**Supplementary Figure 25. In-situ study in sheep cadaver. a-b** Photograph of the sheep left shoulder with an osseosurface electronic device attached on the surface of the humerus. **c-d** Temperature profiles wirelessly recorded from different depths in the sheep cadaver: ~3 cm (**c**) and > 5 cm (**d**).

**
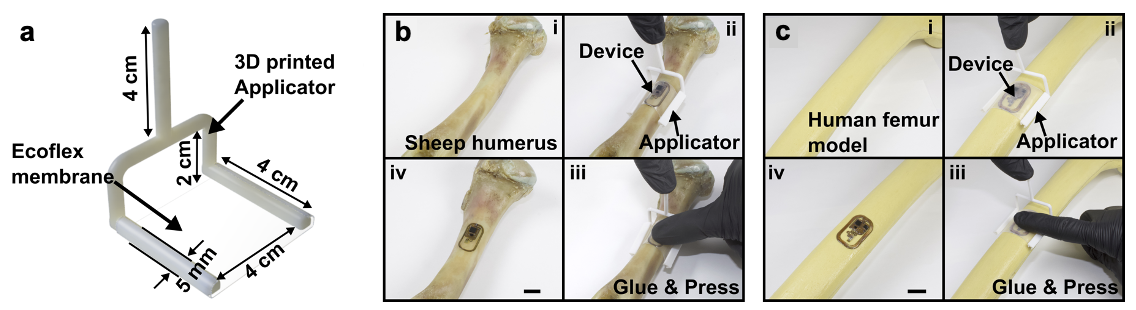
Supplementary Figure 26. Strategy for attaching devices on the bone. a** 3D rendering of the applicator with dimensions suitable for the sheep humerus and devices with footprint of 2.5 cm x 1.5 cm. **b** Step-by-step procedures of attaching a small footprint (1.5 cm x 1 cm) device on the sheep humerus. **c** Step-by-step procedures of attaching a device (2.5 cm x 1.5 cm) on the human femur model.

**
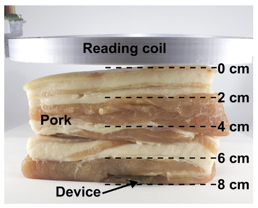
Supplementary Figure 27.** Photograph of the setup for measuring the power harvesting capability and wireless data communication.

**Supplementary Table 1. Record of device implant and failure mode analysis.**

| **Device** | **Implantation Period (days)** | **Device Failure Mechanism** |
| --- | --- | --- |
| 1 | 14 | Data recording was inconsistent with unknown device failure. (Unknown failure) |
| 2 | 6 | Animal died under anesthesia |
| 3 | 28 | Induced pre strain during surgery resulted in out-of-range strain recording. Device was recovered for analysis |
| 4 | 21 | Device re-positioned and device body folded on itself while implanted resulting in diminished RF performance. Device was recovered for analysis |
| 5 | 22 | Encapsulation failure |
| 6 | 43 | High stresses in the tabs holding the device body showed wear and cracking in Parylene encapsulation resulting in circuit damage. |
| 7 | 5 | Animal death due to undetermined cause. |
| 8 | 25 | Defect in encapsulation on self-similar serpentine. |
| 9 | Alive | Animal still alive |
| 10 | 27 | Device recovered for SEM bone attachment analysis. |
