# Supplementary material for: Osseosurface electronics—thin, wireless, battery-free and multimodal musculoskeletal biointerfaces
Source: Nat Commun. 2021 Nov 18;12:6707. doi: 10.1038/s41467-021-27003-2 (PMC8602388; doi:10.1038/s41467-021-27003-2)
Supplement: Supplementary file 3 — Description of Additional Supplementary Files. [file 41467_2021_27003_MOESM3_ESM.pdf]

Description of Additional Supplementary Files

File name: Supplementary Video 1

Description: Video of light delivery with biointerface implantation location on the rat femur.

File name: Supplementary Video 2

Description: Video with corresponding plot of wireless recording of strain of the left femur during gait.

File name: Supplementary Video 3

Description: Video of real time smartphone recording of strain on a sheep humerus cadaver.

File name: Supplementary Video 4

Description: Video of real time smartphone recording of strain on a human femur model.
